# Evolution of pulmonary hypertension in interstitial lung disease: a journey through past, present, and future

**DOI:** 10.3389/fmed.2023.1306032

**Published:** 2024-01-17

**Authors:** Ahmad Arslan, Jorden Smith, Muhammad Raheel Qureshi, Askin Uysal, Kapil K. Patel, Jose D. Herazo-Maya, Debabrata Bandyopadhyay

**Affiliations:** Division of Pulmonary, Critical Care and Sleep Medicine, University of South Florida, Tampa, FL, United States

**Keywords:** pulmonary hypertension, interstitial lung disease, WHO group 3, inhaled treprostinil, screening, pathophysiology, pathobiology

## Abstract

Interstitial lung diseases (ILD) are a spectrum of disorders often complicated by pulmonary hypertension (PH) in its course. The pathophysiologic mechanism of WHO group 3 PH is different to other forms of PH. The advent of PH is a harbinger for adverse events like mortality and morbidity, implying that the PH component of disease expedites deteriorated clinical outcomes. In fact, WHO group 3 PH due to ILD has the worse prognosis among all groups of PH. Hence, early detection of PH by a comprehensive screening method is paramount. Given considerable overlap in clinical manifestations between ILD and PH, early detection of PH is often elusive. Despite, the treatment of PH due to ILD has been frustrating until recently. Clinical trials utilizing PAH-specific pulmonary vasodilators have been ongoing for years without desired results. Eventually, the INCREASE study (2018) demonstrated beneficial effect of inhaled Treprostinil to treat PH in ILD. In view of this pioneering development, a paradigm shift in clinical approach to this disease phenotype is happening. There is a renewed vigor to develop a well validated screening tool for early detection and management. Currently inhaled Treprostinil is the only FDA approved therapy to treat this phenotype, but emergence of a therapy has opened a plethora of research toward new drug developments. Regardless of all these recent developments, the overall outlook still remains grim in this condition. This review article dwells on the current state of knowledge of pre-capillary PH due to ILD, especially its diagnosis and management, the recent progresses, and future evolutions in this field.

## 1 Introduction: who is the accomplice?

WHO Group 3 Pulmonary Hypertension (G3PH) is pulmonary hypertension (PH) that occurs in the setting of chronic lung disease and is the second leading cause of PH after left sided heart failure (1). The most recent World Symposium on Pulmonary Hypertension (WSPH) in 2018 defined group 3 PH as an elevation of mean pulmonary artery pressure (mPAP) ≥20 mmHg in combination with pulmonary vascular resistance (PVR) ≥3 wood units (WU) and a pulmonary capillary wedge pressure (PCWP) ≤15 mmHg confirmed by right heart catheterization (RHC) in a patient with underlying chronic lung disease. However, an updated definition of PH has been proposed in the most recent ESC/ERS guidelines (Table 1). Traditionally, when mPAP rises above 35 or 25 mm of Hg with an associated decrease in cardiac index (CI) to <2 L/min/m^2^, G3PH is defined as severe (1, 2). Table 1 describes the evolution of definition of PH in recent times.

**TABLE 1:** Evolution of pulmonary hypertension definition.

2015	2018	2022
ESC/ERS (27)	WSPH (2)	ESC/ERS (28)
mPAP ≥ 25 mmHg PAWP ≤ 15 mmHg	mPAP > 20 mmHg PAWP < 15 mmHg PVR > 3WU	mPAP > 20 mmHg PAWP < 15 mmHg PVR > 2 WU

mPAP, mean pulmonary artery pressure; PAWP, pulmonary artery wedge pressure; PVR, pulmonary vascular resistance; WU, wood units.

Group 3 pulmonary hypertension (G3PH) is caused by a wide variety of parenchymal pathologies that are commonly classified into obstructive and restrictive diseases based on patterns of pulmonary function tests (PFT). While the obstructive diseases are mostly represented by chronic obstructive pulmonary disease (COPD), the restrictive diseases are further classified into idiopathic interstitial pneumonias (IIP), environmental and occupational lung diseases, connective tissue diseases (CTD-ILD). All the ILDs and any condition leading to chronic hypoxia, including sleep related breathing disorders and chronic exposure to high altitude make up G3PH (3).

The G3PH has a far worse prognosis than PAH (group 1) with 1-, 3-, and 5-year survival of 79.5, 52.7, and 38.1%, respectively, in the former group. In contrast, 1-, 3-, and 5-year survival is 88.2, 72.2, and 59.4%, respectively, in group 1 PAH. The survival rate is even worse in ILD than COPD among G3PH (4). The 1-, 3- and 5-year survival was 71.9, 40.3, and 22.5% in ILD patients compared to 87.7, 66.3, and 54.0%, respectively, in COPD (4).

In spite of poor outcomes, the management of pulmonary hypertension related to interstitial lung disease (PH-ILD) has been disappointing until recently. Unfortunately, the PH-specific therapies studied in PH-ILD have yielded inconsistent results, some even showing harm. However, the recently concluded INCREASE trial has transformed the landscape of diagnosis and management of these patients (5). The current review specifically focusses on G3 pre-capillary PH in relation to ILD- epidemiology, pathophysiology, screening procedures and treatment.

## 2 Epidemiology: a common consort

Prevalence of PH in ILD varies widely in the studies, based on population studied and how PH was defined. The prevalence has been reported as high as 86%, however, this estimate varies between different types of ILD (6). The majority of prevalence data is derived from idiopathic pulmonary fibrosis (IPF), the most common ILD. The reported prevalence of PH among IPF patients ranges between 8 and 15% in early stages to more than 60% at later stages, during lung transplant evaluation (7). As stated earlier, non-invasive screening measurements for PH like echocardiographic or CT chest parameters may have impacted accuracy of estimation.

Data are emerging on prevalence of PH in other ILD entities as well, but limitation of those analysis include small cohorts. A PH prevalence of 31.4% noted in 35 patients with idiopathic non-specific interstitial pneumonia (iNSIP) (8). Similarly, PH was confirmed by RHC in 44% in a cohort of 50 chronic hypersensitivity pneumonitis (HP) patients (9). A 2021 cross-sectional study performed at a tertiary care center in India, showed 46% of 239 ILD patients (32.2% HP, 31.4% CTD-ILD, 36.4% other ILD diseases) had screening echocardiograms suggestive of PH (mPAP estimate > 20 mm Hg) at the time of diagnosis (10). Patients with CPFE are especially susceptible to development of PH with a prevalence of 30–50% (11). Importantly, data from many single center analyses revealed a higher prevalence of PH among CTD-ILD and chronic HP patients in absence of any pertinent clinical feature.

Moreover, PH can complicate clinical courses of rare ILDs such as LAM and sarcoidosis. In the latter, PH is more prevalent in fibrotic sarcoidosis (20%) than non-fibrotic phenotype (12). The exact prevalence in some of the ILD states is difficult to estimate, owing to the rarity of those conditions.

Clinically, PH is a dreadful complication of a wide spectrum of ILD. Economically speaking, chronic lung disease patients with pulmonary hypertension pose a much higher healthcare cost when compared to patients without pulmonary hypertension ($44,732 vs. $7,051) (13). Unfortunately, its diagnosis is still largely confined to large tertiary centers, mainly in those referred for lung transplant. As our clinical practice evolves with screening for PH in ILD patients, the prevalence will most certainly increase.

## 3 Clinical impact and disease outcome: an unwanted guest

It is universally accepted that the advent of PH in ILD patients heralds a poor outcome. The studies are inconsistent in terms of which parameter, mPAP or PVR, in RHC better predict future adverse events, possibly due to small sample sizes (14, 15). It is also well known that RV dysfunction determines the outcomes in all forms of PH. In a single center analysis of 122 patients, G3PH had worse RV dysfunction and poor survival than PAH. However, the interpretation may have been influenced by higher male sex, more comorbidities, and less use of pulmonary vasodilators in the G3PH cohort (16). The same group later demonstrated RV dysfunction (Right ventricle Fraction area change, RVFAC < 28%) in G3PH patients are associated with poor outcomes (17). Because of gradual increase in PVR over time, RV hypertrophy and diastolic dysfunction predominates in early stage with mild ventilatory impairment, as RV gets time to adapt to higher pressure (1, 18). However, persistent hypoxia in chronic lung disease eventually leads to direct RV injury and further elevation of RV afterload, causing RV systolic dilatation and dysfunction leading to “cor-pulmonale” at a later stage of the disease with moderate to severe ventilatory dysfunction (19).

A retrospective study has shown G3PH has worse survival compared to other forms of PH, although the study included all types of G3PH including COPD (20). Patients with ILD complicated by PH carry a worse prognosis with increased need for supplemental oxygen, reduced functional status and decreased survival when compared to ILD patients without PH (21). Lettieri and colleagues noted that IPF patients with PH have significantly lower 6-min walk distance (6MWD) and desaturation than without PH (22). Registry data from French registry and COMPERA reveal PH-ILD patients are more likely to be in New York Heart Association (NYHA) Functional class IV, need for supplemental oxygen and significantly reduced 6MWD than group 1 PAH (4, 21). A single center study showed that the presence of PH is more likely to be associated with increased incidence of acute exacerbations in IPF patients, as well (23).

Besides morbidity, PH-ILD is also associated with worse mortality. In a cohort study of 171 ILD patients, Picarri et al. demonstrated a decreased 3-year survival rate of ILD-PH patients. The cohort showed a survival of 32% in borderline PH (mPAP 21–24 mmHg, PVR ≥ 3 WU), 28% in mild-moderate PH (mPAP 25–35 mmHg, CI ≥ 2 L/min/m^2^), 33% in severe PH (mPAP ≥ 35 mmHg or ≥ 25 and CI ≤ 2 L/min/m^2^). Risk of mortality in this study significantly correlated with mPAP (HR 2.776, 95% CI: 2.057–3.748, *p* < 0.001) (24). Nadrous and co-workers demonstrated a survival of 0.7 years in IPF patients with transthoracic echo showing sPAP greater than 50 mm of Hg and 4 years in those with sPAP less than 50 mm of Hg (7). Another retrospective study of 79 IPF patients who underwent RHC had shown a 1-year mortality of 28% with PH against 5.5% without PH (22). The high PVR predicted 1-year mortality in diffuse lung disease after correction for variables age, gender, diagnosis of IPF, composite physiological index (CPI), in another retrospective analysis of 50 patients (14). The presence of PH portends functional limitations and poor survival in CPFE patients, as well. Considering the adverse outcome, the development of PH in ILD patients is recommended for consideration of lung transplantation in the 2021 consensus document of ISHLT (25).

Nevertheless, PH can be present at early stage of diagnosis of ILD and a slight increase in mPAP above the threshold of 20 mm of hg decreases survival in a multivariate analysis (15). Mild-to-moderate PH can coexist in early stage of ILD and PAP, PVR may not always correlate with parenchymal lung disease (26). In some cases of ILD and PH, the PH may be “disproportionate” to respiratory impairment due to ILD, as evidenced by FVC > 70% of predicted and minimal lung disease on imaging. They may represent a phenotype of coexisting PAH due to predominant pulmonary vascular remodeling. Those patients should be evaluated in PH-specialized center for consideration of treatment with currently available pulmonary vasodilators like PAH.

It is worth noting that much of the currently published data surrounding the epidemiology and prognosis of PH-ILD was collected prior to publication of the 2018 WSPH expanded definition, where PH was defined as mPAP > 25 mm of hg and no mention of PVR (Table 1). It is unclear if application of 2018 WSPH or even 2022 ESR/ESC definition of PH would confer a higher prevalence of PH-ILD or alter its prognosis (3). Establishing a multicenter registry of screening for PH-ILD with longitudinal follow-up data will be valuable to fulfill this unmet need.

## 4 Pathobiology and genetics: what lurks beneath?

The pathobiology of PH-ILD is not completely understood. The common pathogenic mechanism is hypothesized to be a combination of hypoxic pulmonary vasoconstriction and loss of pulmonary bed due to fibrosis.

Much of the evidence of development of pulmonary vasculopathy in chronic lung diseases comes from hypoxia induced animal models. The research demonstrates an elaborate interplay between transforming growth factor B1 (TGF-B1), hypoxia inducible factor 1a (HIF 1a), fibroblast growth factor 2 (FGF2), and vascular endothelial growth factor A (VEGFA) causing an imbalance between the pro- and anti-angiogenic pathways in response to hypoxia (1, 29). Study with hypoxia induced pulmonary hypertension in rats demonstrated dynamic expression of HIF 1a and TGF-B1 with subsequent increase in mPAP and development of RV hypertrophy (30). Additionally, FGFs are well known mediators of pulmonary artery endothelium and smooth muscle cell proliferation, and their levels were noted to be elevated in the animal models of hypoxia (31).

**FIGURE 1:**
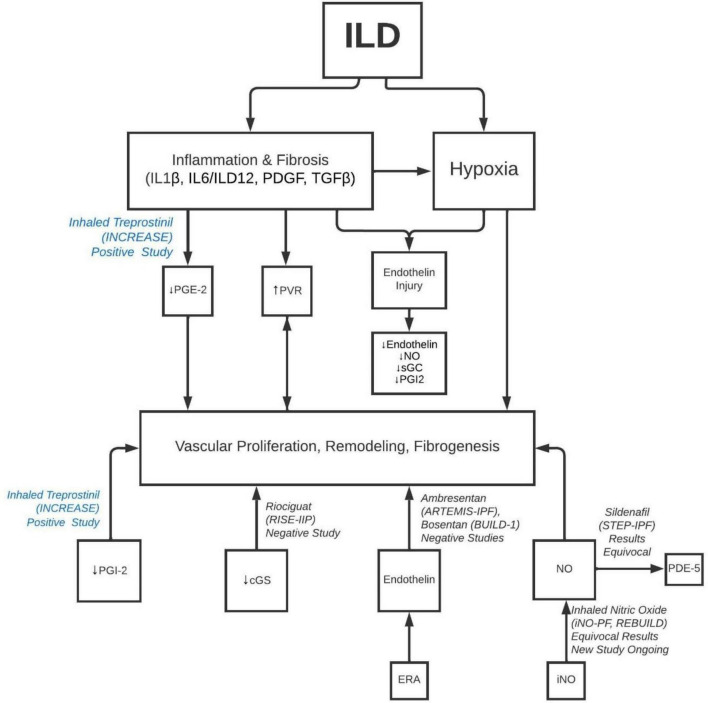
Pathobiology of development of PH in ILD and molecular targets for different classes of PH drugs. ILD, interstitial lung disease; IL, interleukin; PDGF, platelet derived growth factor; TGF, transforming growth factor; PG, prostaglandin; PVR, pulmonary vascular resistance; NO, nitric oxide; sGC, soluble guanylate cyclase; iNO, inhaled nitric oxide; PDE, phosphodiesterase inhibitor.

Histopathologically, this disease was attributed to fibrotic destruction of the pulmonary vascular bed, causing chronic hypoxia, arterial vasoconstriction, and decreased ability of the pulmonary vasculature to accommodate increased cardiac outputs (32). Daton et al. examined 38 sets of explanted lungs from patients with ILD- 7 without PH, 13 with mild to moderate PH, 18 with severe PH (32). The lungs showed a wide variety of histopathological findings, ranging from grade 1 to 4 of Heath and Edwards’s scheme (33) for hypertensive pulmonary vascular disease with progressive structural changes. Interestingly, there was no correlation between the clinical severity of PH and the severity of pulmonary arterial vasculopathy (32). This lack of correlation can further be supported by another study of the histologic features of pulmonary vasculature from patients with PH-ILD and ILD without PH. Reffenach, et al concluded that, while ILD patients without PH demonstrated increased pulmonary vascular wall thickness in the areas of fibrosis, patients with PH-ILD demonstrated a pan vasculopathy with increased vascular wall thickness seen throughout fibrotic and non-fibrotic areas of the lung (29, 34). These findings imply that there is likely an underlying histologic mechanism, i.e., fibroproliferation, that targets the pulmonary vasculature in a way similar to that of parenchyma (Figure 1).

A new insight has been developed in understanding pulmonary vasculopathy with growing focus on microRNAs, small non-coding RNA which moderate gene expression by controlling the translation or inhibiting the degradation (1). Brock et al. showed protective effect of inhibition of miR-20a with improvement in bone morphogenic protein receptor 2 (BMPR2) expression, leading to reduced luminal occlusion of small pulmonary arteries in mouse models (35). It is unclear if this association is confined to PAH only or can be seen in PH due to chronic lung diseases as well. Pulmonary endothelial dysfunction and vascular remodeling was noted in severe hypoxia induced pulmonary hypertension in heterozygous BMPR2 mutant mice (36). In bleomycin-treated rats, persistent downregulation of BMP9/BMPR2/SMAD signaling pathway triggered severe loss of pulmonary arterial endothelium and subsequent pulmonary arterial vascular remodeling, contributing to the development of PH (37). Further research is needed to uncover the role of a common underlying mechanism involving BMPR2 pathway in G3PH.

In addition, research has identified certain genetic imprints in G3PH. A variant of kinase insert domain receptor (KDR), which encodes for VEGFR2, has been shown to correlate with impaired gas transfer. It has been found in older populations with parenchymal lung diseases (38). Patients with IPF also have a unique genetic signature including expression of mediators of pulmonary artery smooth muscle cell (PASMC) and endothelial cell proliferation, Wnt signaling, complement system activation and extracellular matrix (ECM) remodeling and apoptosis (1). Such genetic impression is seen in IPF with and without PH suggesting the role of an exogenous factor in development of PH in predisposed individuals.

Further research is ongoing to unravel the understanding of the complex pathophysiologic mechanism and origin of common etiopathogenic fibroproliferative pathway of the two conditions.

## 5 Screening/diagnosis: a spiritual awakening

The paradigm of G3PH landscape has evolved in recent times. The advent of PH in ILD affects disease trajectory significantly and heralds a poor prognosis. While PH is more common in advanced ILD, it is important to remember PH can be present in mild disease as well. Hence, early detection is essential for prompt diagnosis and treatment. The early manifestations of PH can be subtle and difficult to detect in the presence of ILD. The overt clinical signs of PH appear in the advanced stage of the disease. Hence, a high index of suspicion is crucial for better understanding of clinical presentation.

Unfortunately, no formal screening guidelines exist at present. A recent Delphi study established 9 symptoms that should trigger PH screening: syncope, jugular venous distention, peripheral edema, ascites, abnormal heart sounds, hepatomegaly, dizziness, palpitations, and history of pulmonary embolism. The panel suggested certain objective data should prompt screening: CT evidence of right ventricular enlargement, pulmonary artery enlargement or flattening of the interventricular septum; PFT evidence of DLCO < 40%, rapid decline in DLCO (≥ 15%) or disproportionate DLCO in comparison to FVC (FVC/DLCO > 1.6) or worsening 6MWD in the setting of stable PFTs. A BNP > 300 or NT-proBNP > 395 and certain echocardiographic findings (elevated right ventricle systolic pressure (RVSP), RV enlargement, and low tricuspid annular plane systolic excursion [TAPSE]) were indicators for RHC to confirm PH (39). Although collectively this may be a useful approach, the validity of these parameters has not yet been adequately studied.

The role of pulmonary function test may be useful in assessing PH in ILD patients. In IPF-PH patients, who underwent RHC for lung transplant evaluation, a combination of DLCO < 40% of predicted and hypoxia had a positive predictive value of 87% for diagnosis of PH. On the contrary 20%, who did not fulfill any of these criteria, also had PH (22). Percentage predicted FVC/DLCO ratio, as predictor of PH has been studied principally in scleroderma related ILD. The principal behind this is the disproportionate decrease in DLCO in the presence of PH. Subsequently this model, as predictor for PH, has been validated in other ILDs including IPF, sarcoidosis associated fibrosis (12, 40, 41). The threshold value ranges between 1.4 to 2.2 in various studies (26). However, the utility of this ratio would be limited in CPFE patients in predicting PH. Sobiecka and colleagues noted TLC/DLCO index >1.67 in ILD patients have a high likelihood of PH (42).

Six-min walk test (6MWT) can differentiate exercise performance in PH-ILD vis-à-vis ILD patients without PH. The variables measured are distance walked in the test (6MWD), heart rate recovery (HRR), oxygen saturation and Borg’s dyspnea score. Oxygen desaturation, slower first minute HRR during 6MWT (<13 bpm) provide vital clues for presence of PH. The correlation of 6MWD in ILD patients is unclear (26). Likewise, many other parameters have been proposed in CPET such as delta ETCO_2_ to predict PH-ILD, but they lack sufficient validation (43).

Several radiological features on CT chest should raise suspicion of PH. Most well studied are pulmonary artery (PA)/ascending aorta (AA) ratio >1 and pulmonary artery diameter more than 29 mm. However, these signs have high sensitivity but low specificity in presence of fibrotic lung diseases, because of fibrotic pull of PA (26, 44). Other CT features that can raise suspicion of PH are increased RV size (RV/LV ratio >1), septal flattening.

The role of biomarkers BNP or NT-Pro-BNP has been studied as a screening tool to predict PH. A BNP cut of value of 39.83 ± 4.45 pg/ml has been proposed. Its positive predictive value is limited by confounders such as renal or cardiac dysfunction. Normal BNP/NT-proBNP has a high negative predictive value but does not rule out presence of PH entirely. The utility of this biomarker improves when combined with echocardiographic parameters such as RVSP (45).

Echocardiogram remains the most vital screening tool for PH. The 2022 ESC/ERS guidelines recommend measuring TR jet velocity (TRV) and assessing several other echocardiographic parameters for probability of PH (28). The diagnostic accuracy of echocardiogram to determine systolic pulmonary artery pressure (SPAP) in presence of ILD is less sensitive, hence should not be used alone as screening tool. Besides TRV being an operator dependent variable, the estimation is influenced by altered cardiac position in RV dilatation and fibrosed lung impacting precise measurement of velocity time-integral of tricuspid regurgitant jet. Arcasoy et al reported only a moderate correlation between echocardiogram and RHC measured PAP in chronic lung diseases (46). More recently Keir and colleagues evaluated the utility of screening echocardiogram in 265 ILD patients. The authors found that the 2015 ESC/ERS recommended TRV threshold (peak TRV > 3.4 or 2.9–3.4 m/s with associated features) has higher positive predictive value for detection of PH (86%); surprisingly 40% confirmed PH on RHC were misclassified as “low probability (TRV < 2.8 m/s)” on echocardiogram (47). It was of limited value in excluding PH. Ruocco and coworkers proposed a model for RHC in ILD patients based on BNP > 50 pg/ml, DLCO < 40% of predicted, RVSP > 40 mm of Hg and TAPSE on echocardiogram. However, this application lacks external validation in a prospective trial (48).

Historically, the costs of screening and risks of confirming PH-ILD with RHC have outweighed the benefits, especially since there were no treatment options available (49). The diagnosis of PH by non-invasive methods were subject for debate when the 6th WSPH suggested individualized care at expert PH centers for patients with severe PH-ILD. The expert committee suggested that agents approved for PAH-specific therapy can occasionally be used off-label in these patients, based on expert discretion (2). Nevertheless, clinical practice has significantly evolved since then.

**FIGURE 2:**
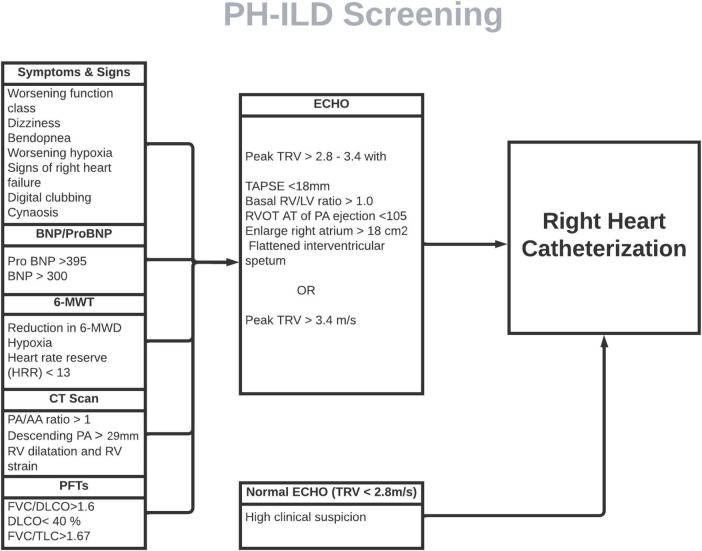
Proposed screening model for PH-ILD screening. ILD patients screened for pertinent signs and symptoms of PH every clinical visit. If clinical features and/or objective data (PFTs, imaging and 6-MWT) suggestive of PH, ECHO should be ordered, followed by RHC if echo is indicative of PH. In small subset of patients in whom clinical features and objective data is strongly suggestive of PH but ECHO is normal, RHC should still pe pursued. BNP, beta-natriuretic peptide; MWT, minute-walk test; MWD, minute-walk distance; PA, pulmonary artery; RV, right ventricle; FVC, forced vital capacity; DLCO, diffusion capacity of lungs for carbon monoxide; TLC, total lung capacity; TRV, tricuspid regurgitation velocity; TAPSE, tricuspid annular plane systolic excursion; RVOT AT, right ventricle outflow tract acceleration time.

Currently, many ILD and PH experts agree that early screening and diagnosis of PH-ILD would be beneficial with advent of new therapeutic option. Many variables have been proposed in conjunction with clinical judgment for early detection PH in ILD patients. Yet, those non-invasive parameters lack high enough sensitivity or specificity to reliably differentiate PH-ILD from ILD. A few composite models have been proposed for recognition of PH in ILD by utilizing variables such as age, BNP, 6MWD, hypoxia, PFT and echocardiographic parameters. (48). However, their utility is limited by small number of study subjects and lack of external validation. A combination of multiple non-invasive screening tests can be utilized to develop a point-based screening system to sufficiently detect PH-ILD. A comprehensive validated objective screening modality, similar to DETECT score in scleroderma, with adequate sensitivity and specificity is the need of the hour (Figure 2).

Many experts agree that echocardiography and BNP or NT-proBNP may be sufficient screening tools, but RHC is essential for diagnosis. Interestingly, the upcoming expert consensus document on ILD-PH recommends performing RHC in presence of high clinical suspicion, even if echocardiogram is normal. Hemodynamic assessment with RHC is being increasingly performed in PH-ILD patients since advent of therapy in PH-ILD. In addition, it also helps to detect concomitant post-capillary PH. The threshold of PAP defining PH has evolved over time (Table 1). The clinical trials and current literature review is largely based on 2015 ESC/ERS guidelines. We do not have any real time data on the applicability of 2022 ESC/ERS criteria for hemodynamic definition of PH and its implication. It is worth exploring in future research.

## 6 Management: light at the end of the tunnel!

The development of PH in the setting of ILD confers a large increase in morbidity and mortality. The 5-year survival rate of ILD-PH patients is estimated to be 34%, nearly half of the 5-year survival rate for ILD without PH (3, 50). Hence, there have been multiple attempts in pharmacologic advancements targeting PH component of PH-ILD disease process. Despite poor outcome, the treatment of PH-ILD has been frustrating, until recently.

The pulmonary vasodilator therapies have shown modest and inconsistent benefits in numerous trials over the years. It has long been hypothesized that pulmonary vasodilators can worsen hypoxia by attenuating ventilation/perfusion mismatch in areas of fibrosis. This problem could be overcome by better delivery of the drugs via inhaled route. The success of RCT with inhaled prostacyclin changed the landscape of PH-ILD therapeutics (5).

Some of the early studies of pulmonary vasodilators in ILD evaluated efficacy of these therapies regardless of the presence or absence of PH. These studies were conceptualized by the fact that properties independent of their known vasodilatory effects may impact common pathophysiologic mechanisms of fibro-proliferation leading to fibrotic lung disease as well as PH. However, RCTs with Bosentan, ARMETIS-IPF were largely disappointing (51, 52). Another proof of concept utilized low DLCO as surrogate marker for PH in IPF patients. The pivotal STEP-IPF trial yielded vital information in this regard (53). The third categories are the PH-specific therapy targeting PH.

### 6.1 Pharmacological therapy of PH-ILD

#### 6.1.1 Phosphodiesterase inhibitors a story of romance

Research surrounding treatment of PH-ILD started in 2010 with cyclic guanosine monophosphate specific phosphodiesterase 5 inhibitors that were the cornerstone of PAH treatment. The STEP IPF trial was a double blinded, randomized, placebo-controlled trial of 180 patients testing the hypothesis that sildenafil would preferentially improve blood flow to well-ventilated regions of the lung in patients with advanced idiopathic pulmonary fibrosis, resulting in gas exchange improvement. The primary outcome, an increase in distance of 6MWT by 20% was not met (53). Notably though, there was significant positive impact in secondary outcomes. Patients in the sildenafil group, had significant improvement in percentage of predicted DLCO (*P* = 0.04), partial pressure of oxygen in arterial blood (*P* = 0.02), and arterial oxygen saturation (*P* = 0.05). Later, two other PDE-5 inhibitor studies with sildenafil published in 2018 (54) and 2021 (55) failed to show any significant effect. However, those studies were limited by lack of PH confirmation by RHC (56). The selection bias of potentially including non-PH patients may have produced a type-II error.

In 2021, Behr et al published a multicenter, l double-blind, randomized, placebo-controlled trial of sildenafil added to Pirfenidone vs. placebo and Pirfenidone to determine severity of disease progression in 177 patients with IPF at risk for PH. The disease progression, defined as decline in 6MWT distance, respiratory related hospitalization, or all-cause mortality, was similar between the two groups (55). Similarly, a shorter study of patients with IPF and DLCO < 35% showed no change in quality-of-life improvement at 12 weeks among patients treated with Nintedanib plus sildenafil vs. Nintedanib plus placebo. Yet, the trial did show a trend toward a significantly reduced rate of decline in FVC < 5% and stabilization of BNP in the sildenafil arm (54).

Recently, a retrospective observational cohort study of 128 ILD-PH patients in UK reported an increase in median survival of 2.18 years with PDE5i treatment against 0.94 years in untreated patients, although overall survival was poor, (58% and 7.7% at 1 and 5 years). The survival difference was notably larger (+ 2.55 years) if RV function was normal at PDE5i initiation (57).

Currently there is enough equipoise regarding the utility of PDE5i in G3PH related to ILD patients. This is partially due to deficiency in trial designs and patient selections, potentially including less severe or no-PH patients. Moreover, most of these studies did not define PH on RHC criteria. The real-life data suggests PDE5i may have a beneficial effect on many variables in PH-ILD patients. Currently the therapy should be considered in selected patients only in specialized centers following careful evaluation.

#### 6.1.2 Endothelial receptor antagonists- not ripe for prime time

Endothelin is a vasoconstrictive peptide that acts on smooth muscle within the pulmonary vasculature. Endothelin receptor antagonists have been found to be potent vasodilators which result in decreased pulmonary vascular pressures. BUILD-1 trial randomized 158 patients with IPF and concomitant PH to receive Bosentan (*n* = 74) or placebo (*n* = 84) with a primary end point of increased exercise capacity after 12 months as measured by 6MWT. Results demonstrated no superiority of Bosentan over placebo (51). Similarly, the B-PHIT trial of Bosentan in PH-ILD did not show any benefit in functional capacity, clinical symptoms or hemodynamics (58).

In 2014, the ARTEMIS-IPF trial explored the efficacy of Ambrisentan in 492 IPF patients, 10% of them had PH. Unfortunately, it was terminated after 34 weeks due increased respiratory hospitalizations and disease progression in treatment arm (52). Subgroup analysis stratified by presence of PH showed a similar outcome.

There have been multiple negative trials evaluating the use of ERAs for treatment of PH-ILD. At present, consensus guidelines recommend against the use of ERA for lack of efficacy and potential harm with Ambrisentan.

#### 6.1.3 Riociguat in ILD-PH- not in the red-zone

The Rise-IIP (2020) trial was a double blinded, randomized, placebo-controlled study to evaluate the safety and efficacy of Riociguat in PH related to idiopathic interstitial pneumonia patients. Unfortunately, this study was terminated early due to an unfavorable risk-benefit profile, with serious adverse events and mortality resulting in 37% of the participants in Riociguat group (59).

#### 6.1.4 Nitric oxide in ILD-PH – NO inhalation yet

The iNO-PF trial was a phase 2 randomized, double blinded, placebo-controlled trial studying the safety and efficacy of increasing doses of inhaled nitric oxide in ILD-PH patients. The 23% patients who received iNO demonstrated at least 15% improvement in moderate to vigorous physical activity by actigraphy, compared to placebo group (60). However, phase 3 REBUILD trial failed to meet the primary end point of 6MWT distance in PH-ILD patients (61). However, patient selection did not include PH as an inclusion criterion. Currently another study is planned with ILD-PH diagnosed by RHC.

#### 6.1.5 Prostanoids in ILD-PH- the dream comes true

In 2021, Waxman et al trialed pulse delivery inhaled Treprostinil in a multicenter, randomized, double-blind, placebo-controlled, 16-week trial that enrolled 326 patients with ILD-PH as documented by RHC. Enrolled subjects had a variety of fibrotic lung diseases including idiopathic interstitial pneumonia (44.8%), combined pulmonary fibrosis and emphysema (25.2%), connective tissue disease related-ILD (22.1%) and chronic hypersensitivity pneumonitis (5.8%). The primary outcome of this study was a statistically significant mean difference in 6MWT distance between the Treprostinil and placebo groups of 31.12 m (95% confidence interval [CI], 16.85 to 45.39; *P* < 0.001). The Treprostinil group also demonstrated a reduction of NT-proBNP by 15% from baseline as compared to a 46% increase in placebo (treatment ratio, 0.58; 95% CI, 0.47 to 0.72; *P* < 0.001). Clinical worsening occurred in 37 patients (22.7%) in the Treprostinil group as compared with 54 patients (33.1%) in the placebo group (hazard ratio, 0.61; 95% CI, 0.40 to 0.92; *P* = 0.04 by the log-rank test) (5).

The INCREASE trial brought a glimmer of hope by demonstrating reduction of NT-proBNP levels and increased exercise capacity in patients with ILD-PH who used Treprostinil, a prostacyclin derivative (5). For successful institution of this therapy, it is noteworthy to consider patient education, strategy for dose titration and side effect mitigation. The communication of the entire care team is paramount to the success of continuation of this medication in these patients.

Currently, Treprostinil is the only FDA approved pulmonary vasodilator to treat group 3 PH-ILD in USA. It is hoped that the successful INCREASE trial study will be followed by further advances in this area leading to more therapeutic options, particularly inhalation agents.

Table 2 narrates the summary of drug trials with pulmonary vasodilators in PH-ILD patients.

**TABLE 2:** Summary of trials for drugs in pulmonary hypertension.

References	Study design	Population studied	Therapy	Primary outcome measured	Result
Collard et al. (62)	RCT	IPF n:11	Sildenafil	Increased 6 MW distance.	Improvement of 49 m
King et al. BUILD-1 (63)	RCT	IPF n: 158	Bosentan.	Improvement in 6 MW distance.	No significant difference noted.
Han et al. (64)	R CT	IPF n: 180	Sildenafil.	Improvement in 6 MW distance.	No significant difference noted.
Seibold et al. BUILD-2 (65)	RCT	SSc-ILD n:163	Bosentan.	Improvement in 6MW distance.	No significant difference noted.
Zisman et al. STEP-IPF (53)	RCT	IPF n:180	Sildenafil.	Improvement in 6 MW distance.	No significant difference noted.
King et al. BUILD-3 (51)	RCT	IPF n:616	Bosentan	Time to IPF worsening (decrease in FVC and DLCO or AE of IPF or death).	No significant difference noted.
Raghu et al. Music (66)	RCT	IPF n:178	Macitentan	Improvement in FVC.	No significant difference noted.
Raghu et al. ARTEMIS-IPF (52)	RCT	IPF n:492	Ambrisentan	Time to disease progression: death, respiratory hospitalization or decline in lung function.	Study terminated early due to more harm in the intervention group.
Corte et al. (58)	RCT	IPF/NSIP n:39	Bosentan	Improvement in pulmonary vascular resistance index.	No significant difference noted.
Saggar et al. (67)	PC	IF/NSIP/CPFE/HP n:15	IV treprostinil	Improvement in RHC parameters, 6MWD, PFTs, ECHO, dyspnea, quality of life	Improvement in hemodynamic, PFTs, dyspnea and quality of life.
Kolb et al. INSTAGE (54)	RCT	IPF n: 274	Sildenafil plus nintedanib	Improvement in St. George’s respiratory questionnaire	No significant difference noted.
Nathan et al. RISE-IIP (59)	RCT	IIP n:147	Riociguat	Improvement in 6 MW distance	No significant difference noted. Study terminated early due to increased serious adverse event and mortality.
Nathan et al. (60)	RCT to OLE	Fibrotic ILD n:48	Inhaled nitric oxide	Improvement in moderate/vigorous physical activity	Significant improvement.
Behr et al. SP-IPF (55)	RCT	IPF n:177	Sildenafil plus pirfenidone	Disease progression	No significant difference noted.
Waxman et al. INCREASE (5)	RCT	IIP n:326	Inhaled treprostinil	Improvement in 6 MW distance	Significant improvement noted.
Nathan et al. (68)	*Post hoc* analysis	IIP N:302	High vs. low dose of inhaled treprostinil	Clinical improvement or clinical worsening	Significant clinical improvement with high dose of inhaled Treprostinil.

RCT, randomized control trial; IPF, idiopathic pulmonary fibrosis; MW, minute walk; SSc-ILD, systemic sclerosis; ILD, interstitial lung disease; FVC, forced vital capacity; DLCO, diffusion capacity of lungs for carbon monoxide; AE, acute exacerbation; NSIP, non-specific interstitial pneumonia; CPFE, combined pulmonary fibrosis and emphysema; HP, hypersensitivity pneumonitis; PFT, pulmonary function test; IIP, idiopathic interstitial pneumonia.

### 6.2 Other therapies: neglected siblings

Besides treatment with pulmonary vasodilators, additional measurements should not be overlooked. Antifibrotic therapy can slow progression of lung function decline, likely decrease mortality and the risk of acute exacerbations (69). Immunosuppression in inflammatory ILD also holds similar potential benefit. Smoking cessation should be offered to patients who continue to smoke. Supplemental oxygen is recommended for patients with interstitial lung disease with chronic resting as well as exertional hypoxemia. Chronic hypoxemia induces hypoxemic pulmonary vasoconstriction and pulmonary arteriolar remodeling, contributing to an increased PVR (70). Sleep-disordered breathing is common in patients with ILD and may worsen PH; should therefore be addressed. Fluid and salt restriction, as well as diuretic therapy, is essential to prevent RV volume overload and failure. Pulmonary rehabilitation has a positive impact on functional capacity and quality of life in ILD patients where 6MWD was improved by a mean of 43 ± 3 m and FVC had improved slightly. Similar benefits were noted in a subset of patients who had PH-ILD (71).

#### 6.2.1 Lung transplant: real home run

The development of PH in the setting of ILD is such a significant prognostic factor that the ISHLT considers it a criterion for lung transplant referral and listing (49). Patients should be referred to transplant centers early in the diagnosis.

## 7 Future ideas for research: world is your oyster

**FIGURE 3:**
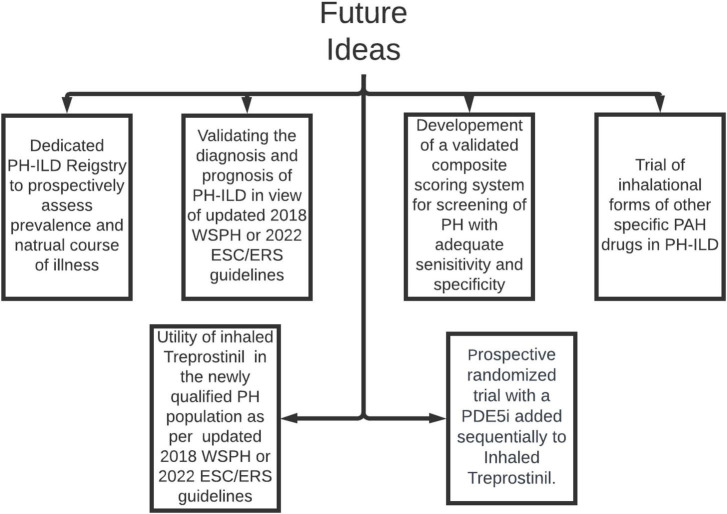
Areas of future research in PH-ILD. PH-ILD, pulmonary hypertension in interstitial lung diseases; WSPH, world symposium on pulmonary hypertension; PAH, pulmonary arterial hypertension; ESC/ERS, European society of cardiology/European respiratory society; PDE5i, phosphodiesterase inhibitor.

In the last few years our understanding of group 3 PH has made tremendous stride, still there are many unfulfilled needs (Figure 3). We feel the community should focus urgently on many aspects of research of this phenotype.

## 8 Conclusion

Interstitial lung diseases identifies a heterogeneous group of conditions characterized by varying degrees of inflammation and fibrosis of lung parenchyma (39, 72). PH affects ILD with increasing prevalence, irrespective of etiology of ILD. The advent of PH in ILD patients’ portent one of the most fatal combinations that we physicians encounter. Hence early diagnosis and treatment is paramount. Unfortunately, due to considerable overlap in signs and symptoms of ILD and PH, detection of PH is often elusive in early stages, before overt clinical signs of RV failure manifests. Consequently, plenty of discussions are focused on screening for PH in ILD patients. A comprehensive screening score is under evaluation. Following a long and arduous journey of unsuccessful drug trials, we have finally achieved success with inhaled Treprostinil to treat this dreadful condition. We hope this novel development will stimulate further research to expand the therapeutic horizons.

## Author contributions

AA: Writing – original draft, Writing – review and editing. JS: Writing – original draft, Writing – review and editing. MQ: Conceptualization, Supervision, Writing – review and editing. AU: Writing – review and editing. KP: Writing – review and editing. JH-M: Writing – review and editing. DB: Conceptualization, Supervision, Writing – original draft, Writing – review and editing.
